# The association between video or telephone telemedicine visit type and orders in primary care

**DOI:** 10.1186/s12911-022-02040-z

**Published:** 2022-11-19

**Authors:** Nathan Juergens, Jie Huang, Anjali Gopalan, Emilie Muelly, Mary Reed

**Affiliations:** 1 Department of Medicine, Kaiser Permanente Oakland Medical Center, Oakland, CA USA; 2 Department of Graduate Medical Education, Kaiser Permanente Oakland Medical Center, Oakland, CA USA; 3 Division of Research, Kaiser Permanente Northern California, Oakland, CA USA

**Keywords:** Telemedicine, Visit type, Primary care, Clinical orders

## Abstract

**Introduction:**

Telemedicine is increasingly relied upon for care delivery in primary care, but the impact of visit type on clinical ordering behavior is uncertain.

**Methods:**

Within Kaiser Permanente Northern California, we identified patients who self-scheduled and completed telemedicine encounters with their personal primary care provider or another available primary care provider in the same medical group, between April 1st, 2020, and October 31st, 2020, while physical distancing restrictions for COVID-19 were in place. We collected patient sociodemographic and clinical characteristics, measures of technology access, and categorized the most common primary encounter diagnoses. We measured proportions of patient-scheduled video versus telephone visits for each of eight diagnosis groups (Skin & Soft Tissue, Musculoskeletal Pain, Back Pain, General Gastrointestinal, Hypertension & Diabetes, Mental Health, Upper Respiratory, and Abdominal Pain), and compared physician orders for medications, antibiotics, lab and imaging studies by visit type within each diagnosis group.

**Results:**

There were 273,301 included encounters, with 86,676 (41.5%) video visits and 122,051 (58.5%) telephone visits. Of the diagnosis groups, Skin & Soft Tissue conditions had the highest proportion of video visits (59.7%), while Mental Health conditions had the highest proportion of telephone visits (71.1%). After adjusting for covariates, the overall rates of medication orders (46.6% vs. 44.5%), imaging orders (17.3% vs. 14.9%), lab orders (19.5% vs. 17.2%), and antibiotic orders (7.5% vs. 5.2%) were higher during video visits as compared to telephone visits (*p* < 0.05). The largest difference within diagnosis groups was for Skin & Soft Tissue conditions, where the rate of medication orders was 9.1% higher than during video visits than telephone visits (45.5% vs. 36.5%, *p* < 0.05).

**Conclusions:**

We observed statistically significant differences in clinician orders by visit type during telemedicine encounters for common primary care conditions. Our findings suggest that, for certain conditions, visual information conveyed during video visits may promote clinical work-up and treatment.

**Supplementary Information:**

The online version contains supplementary material available at 10.1186/s12911-022-02040-z.

## Introduction

Telemedicine has the opportunity to increase access to health care, particularly for those patients with barriers to traditional care models [1, 2]. The availability of high-fidelity digital devices has increased the potential of remote care and has made barriers to widespread implementation less daunting [3]. Today, in many health care organizations, patients are able to interact with medical providers through video and telephone visits or with asynchronous secure messaging.

The shift of health care communication from traditional in-person visits to these relatively novel mediums may impact clinical decision-making. How, and to what degree, physician behavior changes as a result of remote visits has not been extensively studied. The limited data that does exist suggests physicians may prescribe more medicines, particularly antibiotics, during telemedicine encounters than in-person visits for the same diagnoses[4–6]. There is also evidence that adherence to clinical guidelines is less consistent when comparing telemedicine to in-office encounters, a somewhat concerning finding [6, 7].

The influence of the specific telemedicine modality on prescribing behavior has also not been thoroughly investigated. While clinician-specific characteristics have been associated with likelihood of prescription during telemedicine visits, and there have been comparisons of guideline-adherent orders between multiple virtual visit health care companies, the association between video versus telephone encounter on prescription patterns has not been studied [8, 9]. Of note, many studies of prescribing practices during remote encounters have had small sample size and have been conducted on stand-alone, direct-to-consumer telemedicine companies, a model of care that does not foster continuity and which may also influence rates of prescriptions independent of visit modality [6, 7, 10].

The COVID-19 pandemic added urgency and import to the study of telemedicine and its effects on population health. Following the announcement of physical distancing measures, a large proportion of non-urgent outpatient visits shifted to telemedicine. One major medical system reported an 80% decrease in clinic visits and a more than 4000% increase in daily virtual visits over the first weeks of the pandemic [11]. Both patients and providers were essentially forced by the pandemic emergency to rapidly adjust to remote care.

An increasing number of clinical decisions are occurring via telemedicine, but the effect of virtual care visit type on the diagnostics and treatments patients receive is still uncertain [12]. The goal of our study was to observe patients choice of video versus telephone visits for the most common primary care conditions, and to investigate the differences in physician ordering by these visit types. We hypothesized that there would be meaningful differences in orders by visit type and that those differences would vary depending on how important visual information was to the primary condition in question.

## Methods

### Study setting

Kaiser Permanente Northern California (KPNC) is a large integrated health care organization. Since 2016, KPNC patients have been able to schedule primary care appointments through an online patient portal, with the option to choose an in-person, video, or telephone visit. Patients have the option to schedule appointments with their personal primary care provider, or with another available clinician within the study setting. All available providers have experience with and access to the necessary technologies to conduct either telephone or video visits. The option to schedule an in-person office visit was not offered through the online portal after COVID-19 restrictions were enacted in March, 2020, thus patients chose between telephone or video visit when scheduling a primary care appointment.

### Population and measures

**Fig. 1:**
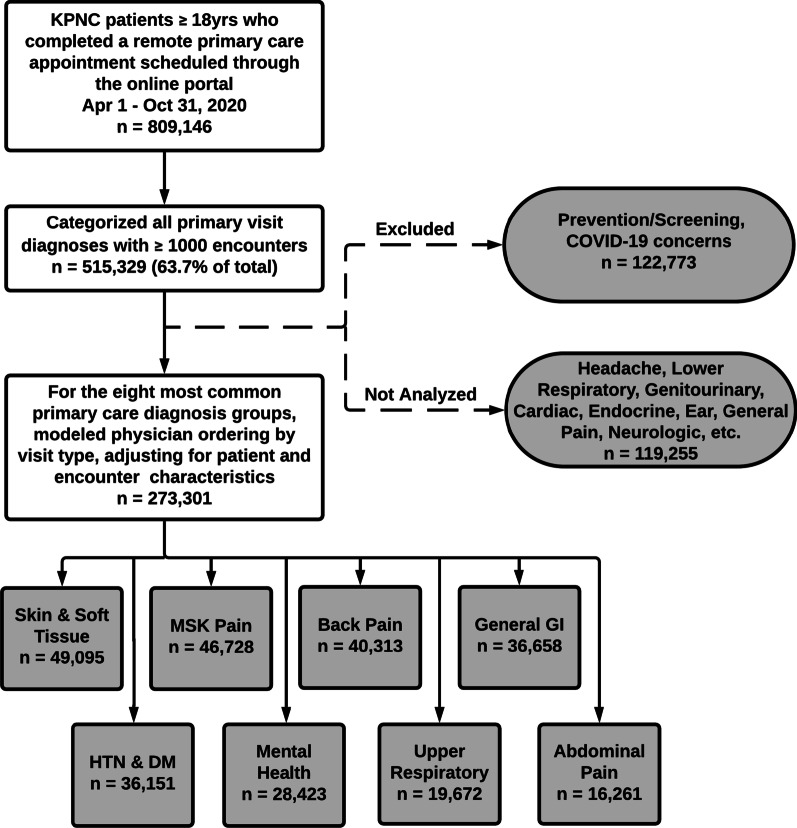
Patient cohort and study design

Using data from the KPNC electronic health record (EHR), we examined all completed video or telephone primary care telemedicine appointments scheduled through the patient portal between April 1st, 2020 and October 31st, 2020 for patients 18 years of age or older (Fig. 1). For each patient who completed at least one telemedicine visit during this interval, we collected sociodemographic characteristics from the EHR (age, sex, race/ethnicity, and English language proficiency). We identified whether patients had completed a video visit or accessed the online patient portal from a mobile device in the prior 365 days, as a measure of technology access. Additionally, based on the patients’ residential addresses, we categorized patients as living in a neighborhood with low socioeconomic status (SES) using 2010 US census block group data. We also categorized patients as living in a neighborhood with greater than or equal to 80% broadband connectivity versus less than 80% connectivity using FCC census tract level data [13].

We acquired patient and visit-level clinical data, including patient comorbidities, the primary encounter diagnosis (using codes from the *International Classification of Diseases, Tenth Revision* (ICD-10)), whether the visit was with a patient’s personal primary care provider (PCP) or a different clinician, the visit type (video or telephone), KPNC medical center through which the visit occurred. Lastly, for each encounter we identified whether there were any associated medicine prescriptions (with an additional indicator for specifically an antibiotic prescription), as well as any associated laboratory or imaging orders.

### Diagnosis groups

To isolate the difference in provider ordering behavior by visit type, we grouped encounter diagnoses by organ system and clinical pathway, with organizational guidance from the *Medical Dictionary for Regulatory Activities* (MedDRA). To focus our analysis on the most common conditions managed in primary care, we initially categorized all the diagnoses in our data set with at least 1,000 associated encounters. This represented 140 unique primary ICD-10 diagnoses and 63.7% of all encounters initially included in the data set. To validate our diagnosis groups, five physicians independently categorized the diagnoses. We measured inter-rater reliability using Randolph’s free-marginal multi-rater Kappa score (overall Kappa = 0.91, 95% CI 0.87–0.94, and within diagnosis groups included in the final analysis Kappa = 0.96, 95% CI 0.92–0.99) [14].

We included the most common eight primary care diagnosis groups in this study. In order of frequency, these were 1- Skin & Soft Tissue, 2—Musculoskeletal (MSK) Pain, 3—Back Pain, 4—General Gastrointestinal (GI), 5—Hypertension (HTN) & Diabetes (DM), 6—Mental Health, 7—Upper Respiratory, and 8—Abdominal Pain (see Additional file 1 for full list of grouped ICD-10 codes). We excluded two common diagnosis groups for methodological reasons. We chose not to analyze prevention and screening visits because this was too broad and diverse a clinical category to interpret differences in ordering by visit-level variables. We also did not analyze visits with a primary diagnosis of COVID-19 or related ICD-10 codes, because these patients were triaged toward specific clinical pathways largely independent of clinician choice and the guidelines for management changed multiple times during the course of our study period.

### Statistical analysis

Within each diagnosis group, we used multivariate logistic regression to examine the association between visit type (video vs. telephone) and clinician orders, adjusting for the sociodemographic, comorbidity and visit-level variables mentioned previously. We included an identifier for patients with repeat encounters for the same primary diagnosis and adjusted for this in our models. We reported adjusted rates calculated by marginal standardization with 95% confidence intervals (CI) [15].

This study was approved by the KPNC Institutional Review Board, and because it only involved retrospective data analysis, the requirement for written informed consent was waived for all patient participants.

## Results

During our study period, there were 809,146 completed adult primary care telemedicine encounters within KPNC. Of these encounters, 482,740 (59.7%) were telephone visits and 326,406 (40.3%) were video visits. There were 273,301 encounters with primary diagnoses included in the final diagnosis group analyses among 172,040 patients. The proportion of each visit type within this sample was comparable to all telemedicine encounters, with 122,051 (58.5%) telephone visits and 86,676 (41.5%) video visits. There were 209,106 (76.5%) encounters completed with a patient’s personal PCP, compared to 64,195 (23.5%) completed with another clinician (Table 1).

**Table 1:** Patient demographics and encounter characteristics by visit type

	%		
	Total	Telephone visit	Video visit
Total	273,301 (*100*)	59.7	40.3
Age, years			
18–40	43.3	42.7	44.2
41–65	41.5	42.0	40.8
> 65	15.2	15.3	15.0
Race/ethnicity			
White	48.3	48.7	47.8
Asian	22.4	19.8	26.4
Hispanic	20.1	21.9	17.4
Black	7.3	7.8	6.6
Multiple/other	1.6	1.6	1.5
Unknown	0.3	0.2	0.3
Sex			
Female	59.3	60.3	58.0
Male	40.7	39.7	42.0
Personal PCP			
Yes	76.5	76.3	76.9
No	23.5	23.7	23.1
Diagnosis group			
Abdominal pain	5.9	6.5	5.1
Upper respiratory	7.2	8.3	5.6
Mental health	10.4	12.4	7.5
HTN & DM	13.2	12.8	13.8
General GI	13.4	15.5	10.3
Back pain	14.8	15.8	13.2
MSK pain	17.1	16.6	17.9
Skin & soft tissue	18.0	12.1	26.6

Percentages shown are column percentages. Additional patient and encounter characteristics were adjusted for in the logistic regression analyses, and can be found in Additional file 1

*HTN* hypertension, *DM* diabetes, *GI* gastrointestinal, *MSK* musculoskeletal

**Fig. 2:**
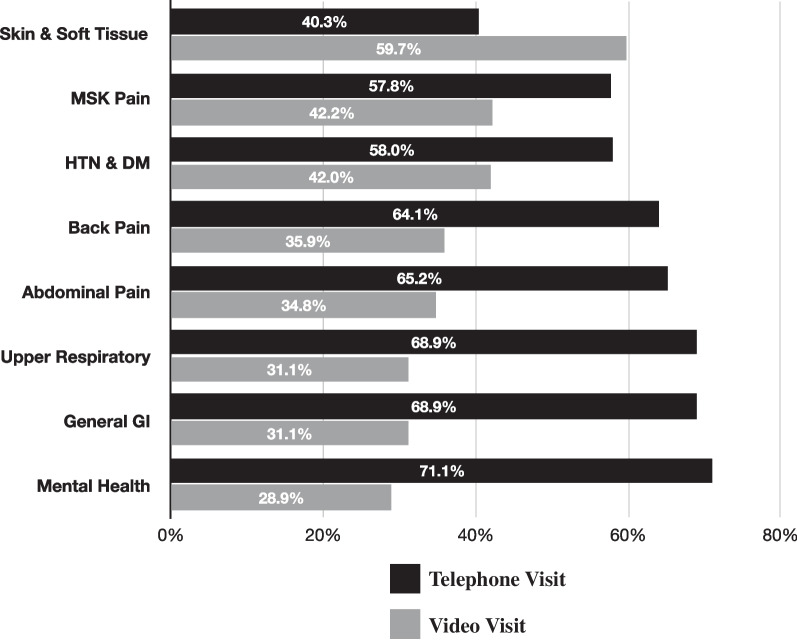
Proportions of telephone and video visits, by diagnosis group. Notes: Percentages shown are within group proportions. Abbreviations: HTN, Hypertension; DM, Diabetes; GI, Gastrointestinal; MSK, Musculoskeletal

The most frequent diagnosis category in our study was Skin and Soft Tissue (18.0%), followed by Musculoskeletal Pain (17.1%), Back Pain (14.8%), General Gastrointestinal (13.4%), Hypertension & Diabetes (13.2%), Mental Health (10.4%), Upper Respiratory (7.2%), and Abdominal Pain (5.9%) (Table 1). Skin and Soft Tissue was the only group with more video (59.7%) than telephone visits, while the other seven groups included majority telephone encounters. Mental Health conditions had the highest proportion of telephone (71.1%) of any group (Fig. 2).

Orders for medications were frequently associated with encounters for Upper Respiratory (59.9%), Mental Health (56.7%), Hypertension & Diabetes (47.1%), General Gastrointestinal (42.8%), and Skin & Soft Tissue (44.7%) conditions, though these groups generated comparatively few imaging orders (2.3%, 1.0%, 4.4%, 3.5%, and 1.6%, respectively). An imaging study was ordered during 36.9% of visits for Musculoskeletal Pain compared to only 18.8% of visits for Back Pain. The majority of diagnosis groups had very low rates (less than 3%) of orders for an antibiotic medicine, with the exception of Upper Respiratory (32.6%) and Skin & Soft Tissue (15.7%) conditions.

**Fig. 3:**
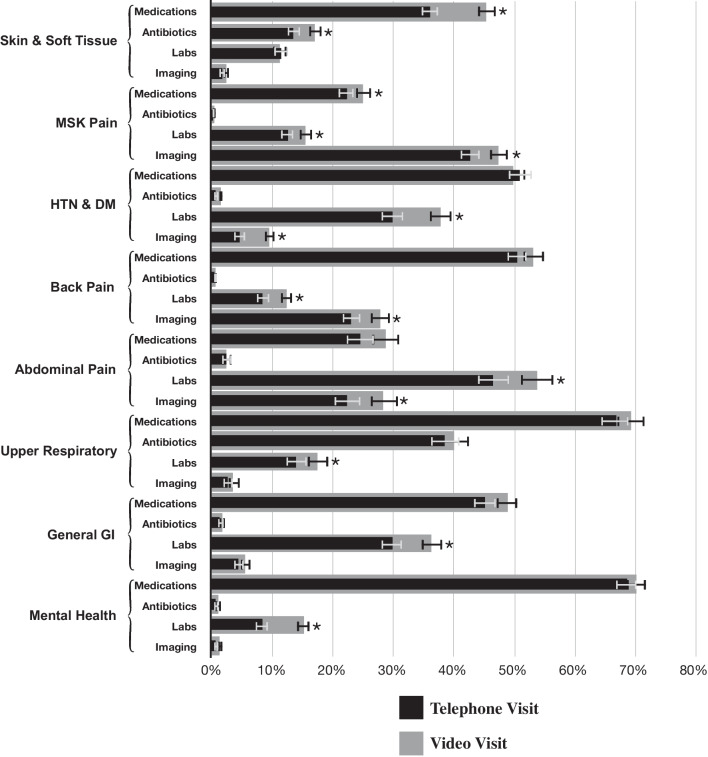
Predicted probabilities of clinician orders for common primary care diagnoses, by visit type. Notes: Percentages shown are predicted probabilities, adjusted for all patient and encounter covariates. Error bars show 95% confidence intervals, and asterisks (*) represent two-sided *p* < 0.05. Abbreviations: HTN, Hypertension; DM, Diabetes; GI, Gastrointestinal; MSK, Musculoskeletal

After adjustment, overall we found video visits were significantly associated with higher rate of clinician orders across each order type assessed, with 2.1% higher rate of a medication order (46.6% vs. 44.5%), 2.3% higher rate of a lab order (19.5% vs. 17.2%), 2.4% higher rate of an imaging order (17.3% vs. 14.9%), and 2.3% higher rate of an antibiotic order (7.5% vs. 5.2%) during video visits compared to telephone visits (*p* < 0.05 for each). Within the diagnosis groups we observed a similar trend. Compared to telephone visits for the same conditions, there were significantly higher rates of medication orders during video visits for Skin & Soft Tissue (45.3% vs. 36.3%) and Musculoskeletal Pain (25.1% vs. 22.4%) conditions, significantly higher rates of lab orders during video visits for Musculoskeletal Pain (15.6% vs. 12.6%), Hypertension & Diabetes (37.8% vs. 30.0%), Back Pain (12.3% vs. 8.5%), Abdominal Pain (53.8% vs. 46.8%), General Gastrointestinal (36.3% vs. 30.0%), and Mental Health (15.1% vs. 8.4%) conditions, significantly higher rates of imaging orders during video visits for Musculoskeletal Pain (47.4% vs. 42.9%), Hypertension & Diabetes (9.6% vs. 4.6%), Back Pain (27.8% vs. 23.2%), and Abdominal Pain (28.4% vs. 22.5%) conditions, and significantly higher rates of antibiotic orders during video visits for Skin & Soft Tissue (17.0% vs. 13.6%) conditions (*p* < 0.05 for each, Fig. 3).

## Discussion

In this study, we present cross-sectional observations on a large cohort of adults accessing care via telemedicine for common primary care conditions during the COVID-19 pandemic. We found video visits were consistently associated with a higher rate of orders compared to telephone visits.

The largest observed difference in rate of orders between video and telephone telemedicine across all diagnosis groups and order types was for medication orders for Skin & Soft Tissue conditions; the diagnosis of dermatologic disease depends on visualizing the patient, so this was a predictable finding. Despite the relatively high rate of antibiotic prescriptions, we did not observe a significant ordering difference between video and telephone visits for Upper Respiratory conditions. This is somewhat reassuring given prior research suggesting an increase in inappropriate antibiotic ordering during telemedicine encounters [6]. With the exception of difference in medication orders for Skin & Soft Tissue and Musculoskeletal Pain, all of the other statistically significant differences in order rate were for lab and imaging orders, which are for the diagnosis or management of disease rather than for treatment. The reasons why video visits may result in additional work-up of disease compared to telephone and the appropriateness of these ordering differences remain unknown and warrant further investigation.

Patient choice of visit type by diagnosis group also offers insights. The group with the highest proportion of video visits was Skin & Soft Tissue, while Mental Health had the highest proportion of telephone visits; again, this is consistent with the type of information required to diagnose and manage these conditions, and it seems patients in our study population understood this when scheduling visits. Musculoskeletal Pain diagnoses had the second highest proportion of video visits of any group, and the musculoskeletal physical exam has also been shown to translate well to remote video visits [16].

To our knowledge, this is one of the only studies investigating the association between video and telephone telemedicine visit type and clinical orders and is certainly the largest in sample size. Given the magnitude, even the modest differences in clinical orders we observed in our cohort represent thousands of medications, labs or imaging studies. Our data do not indicate whether the ordering differences observed were appropriate or not, for example, whether clinicians ordered more medications than indicated during video visits or, alternatively, fewer than indicated during telephone visits; that is outside of the scope of this analysis and should be investigated further in future research. Also, given the period of our study, directly contrasting our findings to orders associated with in-person primary care visits was not feasible, though a prior study by our group on patient encounters between January 2016 and May 2018 found comparable rates of medication, lab and imaging orders [17].

As just alluded to, it should be emphasized that this study occurred during the COVID-19 pandemic, a public health emergency. In order to maintain access to care and prevent exposure to the coronavirus, in March of 2020, the Centers for Medicare & Medicaid Services (CMS) broadened reimbursement for telemedicine [18]. This expanded coverage has been extended through the end of 2021, but it is not yet clear what telehealth services will be reimbursed long-term, in-particular whether CMS will continue to reimburse for telephone visits at the same rate as video visits [19, 20]. In our study, we did observe differences in clinical behavior between video and telephone visits for common primary care diagnoses, though many of the differences were relatively small, and for some order types and diagnosis groups, there were no differences between the two visit modalities. These findings may help inform policymakers when deciding on the future of telemedicine reimbursement.

### Limitations

Our study has several limitations. First, the visits in our dataset were self-scheduled by the patients. This raises the possibility that the differences we observed in order rate by visit type were not due to differences in telecommunication modality, but, rather, because of differences in clinical severity between patients who chose video versus telephone visits. While we adjusted for a large variety of patient and clinical variables and attempted to compare ordering within diagnostic groups with limited ranges of severity, we cannot exclude the possibility of this confounder.

Additionally, our data contain a substantial subset of individual patients with multiple telemedicine encounters. While this is not unexpected given the duration of our study, we know that the rate of clinician orders is likely impacted by repeat visits for the same condition, for example, a subsequent visit monitoring the effect of a new prescription may generate different orders than a follow-up visit to discuss the results of a recent lab or imaging study. We adjust for repeat visits for the same ICD-10 diagnosis in our models, but it is possible that due to coding variability we did not fully capture these longer term episodes of care. Lastly, as with any retrospective data study, the observed associations cannot be used to attribute causation.

## Conclusions

In this study of remote visits for common primary care conditions, we found a subtle but statistically significantly higher in the rate of medication, antibiotic, lab and imaging orders during video visits compared to telephone visits. There was some nuance within diagnosis groups, but the overall trend was consistent. Video visits offer clinicians visual information about their patients while telephone visits do not, and there are many diagnoses and triaging decisions that rely on such visual information.

Physician prescribing behavior is difficult to study; it is a complex behavior dependent on multiple inputs, including personal and patient characteristics, medical experience, health care organization, reimbursement model, trends in scientific literature, as well as the modality by which providers and patients interact.

This study was an initial attempt to examine how different forms of telemedicine may impact clinical behavior in remote primary care. Future research focused on more narrow clinical areas should further explore the specific orders (e.g. abdominal CT scans, blood chemistries, opioid pain relievers) that comprise the differences between video and telephone visits within relevant diagnosis groups. This would allow for a better understanding of whether the ordering differences observed are appropriate and/or guideline concordant.

We found that video telemedicine visits appear to generate more clinical actions than telephone visits, particularly in clinical areas where visual information is likely to be particularly useful. Further examination of clinical usefulness of video communication between patients and physicians is needed to best utilize telemedicine beyond the pandemic period for ongoing primary care practice.

## Supplementary Information

**Additional file 1:** Diagnosis Category ICD-10 Codes.

## Data Availability

The datasets generated and analyzed during the current study are not publicly available as they contain protected health information and individual privacy could be compromised, but can be made available in a de-identified manner from the corresponding author on reasonable request.

## Abbreviations

KPNC: Kaiser Permanente Northern California
EHR: Electronic health record
SES: Socioeconomic status
FCC: Federal Communications Commission
PCP: Primary care provider
ICD: International Classification of Diseases
MedDRA: Medical Dictionary for Regulatory Activities
MSK: Musculoskeletal
GI: Gastrointestinal
HTN: Hypertension
DM: Diabetes
CI: Confidence intervals
