# Supplementary material for: The association between video or telephone telemedicine visit type and orders in primary care
Source: BMC Med Inform Decis Mak. 2022 Nov 19;22:302. doi: 10.1186/s12911-022-02040-z (PMC9675192; doi:10.1186/s12911-022-02040-z)
Supplement: Supplementary file 1 — Additional file 1: Diagnosis Category ICD-10 Codes. [file 12911_2022_2040_MOESM1_ESM.pdf]

Supplemental Materials

S1: Diagnosis Category ICD-10 Codes

Abdominal Pain: "ABDOMINAL PAIN" | "EPIGASTRIC ABDOMINAL PAIN" | "RUQ ABDOMINAL PAIN”

Back Pain: "LOW BACK PAIN" | "CHRONIC LOW BACK PAIN" | "LUMBAR RADICULOPATHY" | "CERVICAL RADICULOPATHY" | "NECK PAIN" | "CHRONIC LOW BACK PAIN W LUMBAR RADICULOPATHY"| "THORACIC SPINE PAIN" | "BACK PAIN" | "LUMBAR MUSCLE STRAIN, INIT"

General Gastrointestinal: "GERD (GASTROESOPHAGEAL REFLUX DISEASE)" |"ABDOMINAL BLOATING" | “DYSPEPSIA"| "IRRITABLE BOWEL SYNDROME" | “NAUSEA"| "DIARRHEA" | "CONSTIPATION" | "GASTROENTERITIS" | "GASTRITIS" | "RECTAL BLEEDING" | "EXTERNAL HEMORRHOID”

Hypertension & Diabetes: "HTN (HYPERTENSION)" | "DM 2" | "PREDIABETES" | "DM 2 W HYPERLIPIDEMIA" | "ELEVATED BP READING WO HTN DIAGNOSIS" | "DM 2 W PERIPHERAL NEUROPATHY"

Mental Health: "ANXIETY" | "INSOMNIA" | "ANXIETY DISORDER"| "STRESS" |"ADJUSTMENT DISORDER W MIXED ANXIETY AND DEPRESSED MOOD" | "GENERALIZED ANXIETY DISORDER" | "ADJUSTMENT DISORDER W ANXIETY" | "MAJOR DEPRESSIVE DISORDER, RECURRENT EPISODE, MODERATE"

Musculoskeletal Pain: "RIGHT KNEE JOINT PAIN" | "LEFT KNEE JOINT PAIN" | "RIGHT SHOULDER JOINT PAIN" | "LEFT SHOULDER JOINT PAIN"| "RIGHT FOOT PAIN" | "LEFT FOOT PAIN" | "LEFT HAND PAIN" | "BILAT KNEE JOINT PAIN" | "JOINT PAIN" | "RIGHT HIP JOINT PAIN" | "LEFT HIP JOINT PAIN" | "RIGHT WRIST JOINT PAIN" | "RIGHT ANKLE JOINT PAIN" | "RIGHT HAND PAIN" | "LEFT ANKLE JOINT PAIN" | "LEFT WRIST JOINT PAIN" | "LEFT LEG PAIN" | "RIGHT LEG PAIN"| "RIGHT KNEE JOINT PAIN"| "LEFT KNEE JOINT PAIN" | "RIB PAIN" | "MYALGIA"

Upper Respiratory: "URI (UPPER RESPIRATORY INFECTION" | "ALLERGIC RHINITIS" | "PHARYNGITIS" | "ACUTE SINUSITIS" | "SEASONAL ALLERGIC RHINITIS" | "THROAT PAIN" | "CHRONIC SINUSITIS"

Skin & Soft Tissue: "SKIN LESION" | "DERMATITIS" | "RASH" | "ACNE" | "CELLULITIS" | "ALOPECIA" | "SUBCUTANEOUS MASS" | "ECZEMA" | "SEBORRHEIC DERMATITIS" | "URTICARIA" | "FOLLICULITIS" | "HERPES ZOSTER" | "CONTACT DERMATITIS" | "PRURITUS" | "SKIN MASS”
